# RNA editing regulates host immune response and T cell homeostasis in SARS-CoV-2 infection

**DOI:** 10.1371/journal.pone.0307450

**Published:** 2024-08-23

**Authors:** Molly Huang, Adam Mark, Jessica Pham, Karina Vera, Amanda M. Saravia-Butler, Afshin Beheshti, Qingfei Jiang, Kathleen M. Fisch

**Affiliations:** 1 Department of Obstetrics, Gynecology & Reproductive Sciences, University of California San Diego, La Jolla, California, United States of America; 2 Bioinformatics and Systems Biology Graduate Program, University of California San Diego, La Jolla, California, United States of America; 3 Center for Computational Biology & Bioinformatics, University of California San Diego, La Jolla, California, United States of America; 4 Division of Regenerative Medicine and Moores Cancer Center, University of California San Diego, La Jolla, California, United States of America; 5 KBR, Space Biosciences Division, NASA Ames Research Center, Moffett Field, California, United States of America; 6 Blue Marble Space Institute of Science, Seattle, Washington, United States of America; 7 Stanley Center for Psychiatric Research, Broad Institute of MIT and Harvard, Cambridge, Massachusetts, United States of America; 8 COVID-19 International Research Team, Medford, Massachusetts, United States of America; Instituto Butantan, BRAZIL

## Abstract

Adenosine to inosine (A-to-I) RNA editing by ADAR1 has been implicated in maintaining self-tolerance, preventing autoimmunity, and mediating antiviral immunity. Foreign viral double-stranded RNA triggers rapid interferon response and activates ADAR1 in the host immune system. Emerging data points to a role of ADAR1 A-to-I editing in the inflammatory response associated with severe COVID-19 disease. We identify A-to-I editing events within human whole transcriptome data from SARS-CoV-2 infected individuals, non-infected individuals, and individuals with other viral illnesses from nasopharyngeal swabs. High levels of RNA editing in host cells are associated with low SARS-CoV-2 viral load (p = 9.27 E-06), suggesting an inhibitory effect of ADAR1 on viral infection. Additionally, we find differentially expressed genes associated with RNA-modifications and interferon response. Single cell RNA-sequencing analysis of SARS-CoV-2 infected nasopharyngeal swabs reveals that cytotoxic CD8 T cells upregulate ADAR1 in COVID-19 positive samples (p = 0.0269). We further reveal ADAR1 expression increases with CD4 and CD8 T cell activation, and knockdown of ADAR1 leads to apoptosis and aberrant IL-2 secretion. Together, our data suggests A-to-I RNA editing is required to maintain healthy homeostasis of activated T cells to combat SARS-CoV-2 infection.

## Introduction

Adenosine to inosine (A-to-I) RNA editing regulates innate immune response in host cells during viral infections [1–3]. Adenosine deaminases that act on RNA (ADAR) are a family of enzymes that is responsible for the editing of double-stranded RNA (dsRNA) of cell intrinsic or extrinsic sources. Foreign positive-strand RNA viruses, such as severe acute respiratory syndrome coronavirus (SARS-CoV-2), produce dsRNA that are readily detected by ADAR1 and other protein components of the antiviral innate immune response and trigger interferon (IFN) production [4, 5].

ADAR1 RNA editing can act in both a proviral and an antiviral manner, depending on the virus and host [3, 6]. ADAR1 RNA editing increases human immunodeficiency virus type 1 expression [7] and enhances vesicular stomatitis virus replication [8], demonstrating a proviral role. Conversely, ADAR1 RNA editing protects against viruses, such as measles [9] and hepatitis C [10], and is thus antiviral. A-to-I RNA editing was readily detected in viral sequences of SARS-CoV-2 from diverse geographic locations [11–14]. RNA-editing in SARS-CoV-2 transcriptome is inversely correlated with patient mortality, suggesting an anti-viral role of ADAR1 [15]. In contrast, an A-to-G mutation was detected in the spike protein (D614G), which greatly enhances the infectibility of SARS-CoV-2 [16], suggesting a pro-viral function of ADAR1.

Other than the editing of the viral transcriptome, the activation of ADAR1 can also induce A-to-I RNA modifications in the host cells. In this scenario, the function of A-to-I RNA editing is to distinguish self-dsRNA from non-self dsRNA from viral invasion to prevent detection by cytosolic dsRNA sensors, such as protein kinase R (PKR) or melanoma differentiation-associated protein 5 (MDA5) [17–19]. Abnormal dsRNA sensing leads to a cascade event, including uncontrolled IFN production, neurosis, and apoptosis of host cells [20, 21]. ADAR1 host RNA editing has been previously observed in whole transcriptome data derived from bronchoalveolar lavage fluids of COVID-19 patients [14, 22] and nasopharyngeal swabs [23]. Interestingly, Saichi et al. found that COVID-19 severity increases with a decrease in ADAR protein expression in COVID-19 host cells [24].

Infection severity and mortality of SARS-CoV-2 infection is likely caused by dysfunction of innate and adaptive immunity [25, 26]. For example, patients with severe COVD-19 demonstrate increased proliferation, activation, and cytotoxicity in CD4+ and CD8+ T cells [27, 28]. As such, another point of interest of ADAR1-induced RNA-editing during SARS-CoV-2 infection is T cell priming through IFN signaling [29]. The role of ADAR1 in adaptive immunity was previously demonstrated by mouse models. Loss of ADAR1 results in excessively expressed IFN-stimulated genes, causing impaired early T cell development, failed negative selection, and autoimmunity [30–32]. However, the functions of host RNA editing in regulation of T cell activation and immune response to SARS-CoV-2 infection are unclear.

**Fig 1:**
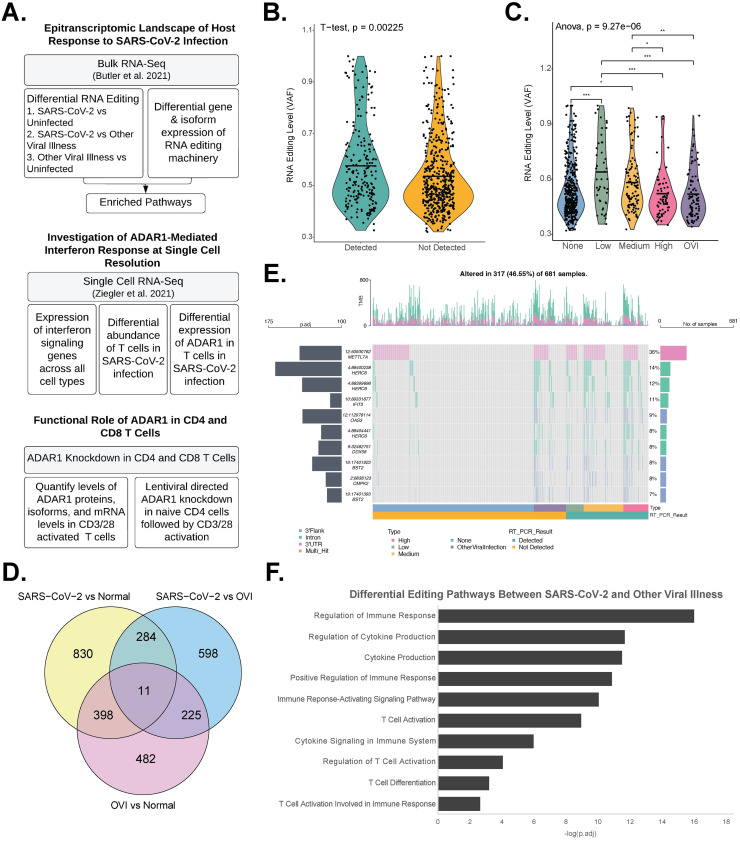
Differential editing and enriched pathways in SARS-CoV-2 nasopharyngeal swab samples. a. Overview of workflow. b. Overall RNA editing levels are significantly higher in SARS-CoV-2 positive patients than negative patients (p = 0.00225). c. RNA editing levels increase with decreasing viral load (p = 9.27E-6). * p < 0.05; ** p < 0.01; *** p < 0.001. d. Venn diagram of differential RNA editing sites found in three comparisons: SARS-CoV-2 positive vs normal: 1,523; other viral illness vs normal: 1,116; SARS-CoV-2 positive vs other viral illness: 1,118. e. Differential editing sites between COVID-19 positive and negative samples are found in introns, the 3’ UTR, and the 3’ Flank. f. Pathway analysis of differentially edited genes between SARS-CoV-2 and other viral illness samples (adj.p < 0.05).

In this study, we examined the relationship between ADAR1-mediated RNA-editing, T cell activation, and immune response in the host cells of SARS-CoV-2 infection (Fig 1A). We report that A-to-I host-editing is elevated in human whole transcriptome data from COVID-19 patient nasopharyngeal swabs [33] and further identify both differentially edited and expressed genes associated with IFN response and T cell activation. Interrogation of COVID-19 positive single cell RNA-sequencing (scRNA-seq) dataset of SARS-CoV-2 infected nasopharyngeal swabs [34] reveals upregulated ADAR1 expression in IFN responsive cytotoxic CD8 T cells. Furthermore, we find that activation of ADAR1 promotes healthy homeostasis in T cell proliferation.

## Results

### RNA editing events are enriched in immune response and T cell activation

Human whole transcriptome data from nasopharyngeal swabs was collected from 215 SARS-CoV-2 positive, 82 positive for other viral illness (OVI), and 429 SARS-CoV-2 negative patients, as described previously [33]. Several trends could be observed between A-to-I RNA editing events and clinical parameters (Fig 1). Overall editing levels were higher in SARS-CoV-2 positive patients than negative patients, as represented by variant allele frequency (Fig 1B, p = 0.00225). Furthermore, the overall RNA editing levels was negatively correlated with viral load, suggesting RNA editing may be important for damping SARS-CoV-2 infection (Fig 1C, p = 9.27E-06). However, this finding may be a result of other possible mechanisms or be due to technical artefacts, such as a lower fraction of human transcriptome sequencing reads in patients with high viral loads, warranting further investigation.

We analyzed the number of differential editing sites in the following comparisons: SARS-CoV-2 positive vs negative: 1,523; OVI positive vs negative: 1,116; SARS-CoV-2 positive vs OVI positive: 1,118; SARS-CoV-2 High vs Low Viral Load: 687 (Fig 1D and S1 Table). 11 differential editing sites were present in all comparisons (S1 Fig in S1 File). Differential editing genes between SARS-CoV-2 vs normal samples were enriched in pathways such as cytokine signaling in immune system (S2 Table, p.adj = 2.00E-06), cytokine production (S2 Table, p.adj = 9.32E-09), and innate immune response (S2 Table, p.adj = 7.42E-14). The top differential editing genes included several IFN signaling genes (ISGs) such as IFIT3, OAS3, and DDX58 (Fig 1E). Of note, type I and type II ISGs–IFNAR1, IFNGR1, and IFNGR2–demonstrated decreased editing in SARS-CoV-2 samples when compared to OVI samples (S1 Table). Notable genes that demonstrated increased editing in SARS-CoV-2 samples when compared to OVI samples include SAMHD1 and hnRNPC (S1 Table). Loss of SAMHD1 was previously found to downregulate SARS-CoV-2 RNA levels in macrophage cell lines [35], and hnRNPC may play a proviral role during SARS-CoV-2 infection [36]. RNA editing events between SARS-CoV-2 positive and control samples were primarily intronic, 3’UTR, or 3’Flank (Fig 1E and S1 Table).

Next, we focused on differential editing between SARS-CoV-2 and OVI to investigate unique ADAR1-regulated pathways in SARS-CoV-2 patient cohort (Fig 1F). Differential editing genes between these two groups were significantly enriched (p.adj < 0.05) in T cell pathways including T cell activation (Fig 1F, p.adj = 1.12E-09), regulation of T cell activation (Fig 1F, p.adj = 9.00E-05), and T cell activation involved in immune response (Fig 1F, p.adj = 0.00237). These pathways were not shared among differential editing in viral responses relative to normal, indicating a differential T cell response between infection of SARS-CoV-2 and OVI (S2 Table).

### Identification of differential expressed RNA-editing targets and immune-related genes

**Fig 2:**
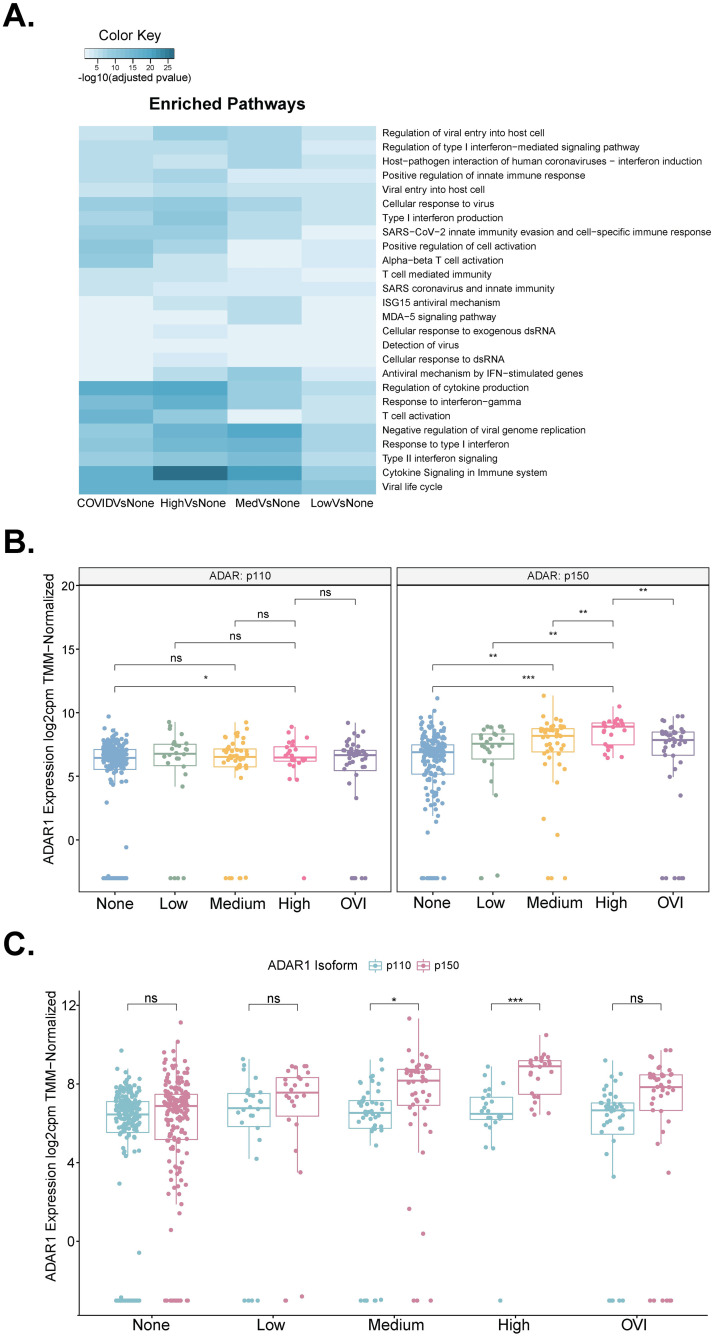
Differentially expressed genes and ADAR isoforms in SARS-CoV-2 infection. a. Pathway analysis performed on differentially expressed genes between viral loads and other viral illness samples demonstrates the enrichment of pathways (Reactome and GO:BP) involved with immune cell response such as T cell activation involved in immune cells, and interferon signaling and responses. b. Gene expression of ADAR1 in COVID-19 viral load divided by isoforms p110 and p150 with a t-test comparison. * p < 0.05; ** p < 0.01; *** p < 0.001; ns: not significant. c. Gene expression of ADAR1 isoforms p110 and p150 divided by COVID viral load with a t-test comparison. * p < 0.05; *** p < 0.001; ns: not significant.

Upon observing elevated A-to-I RNA-editing and enrichment of immune response pathways, we interrogated differential expression of genes during SARS-CoV-2 infection with relevance to differential RNA editing events. A total of 1,505 up-regulated and 1,029 down-regulated differentially expressed genes were found between SARS-CoV-2 positive and control samples (S3 Table). In addition, 171 of these differentially expressed genes were also differentially edited (S3 Fig in S1 File). The simultaneously differentially edited and upregulated genes between SARS-CoV-2 positive and negative samples were consistently IFN-related genes (S4 Fig in S1 File). We confirmed the upregulation of cytokines (CXCL10, CXCL 11, CXCL12, CXCL13, and CCL8) and other mediators of IFN pathways (STAT1, STAT2, and HERC6) [33, 37] between SARS-CoV-2 positive and control cohorts (S3 Table). Further pathway analysis revealed that differentially expressed genes were significantly enriched in dsRNA sensing pathways, such as the RIG-I and MDA5 dsRNA signaling pathways cellular response to dsRNA (Fig 2A). The majority of the pathways were related to immune response, including T cell activation, antiviral mechanism by IFN-stimulated genes, and host-pathogen interaction of human coronaviruses—interferon induction (Fig 2A). These data suggested that RNA editing activity likely contributes to suppression of dsRNA sensing and control of immune response during SARS-CoV-2 infection.

ADAR1 expression was previously found to increase in COVID-19 [23]. As ADAR1 was the only member of the ADAR family that was differentially expressed in the SARS-CoV-2 positive versus negative cohorts (S5 Fig in S1 File), we conclude that host A-to-I RNA-editing due to SARS-CoV-2 infection is primarily attributed to ADAR1 activity (S3 Table, p.adj = 9.42E-27). Notably, ADAR1 has two isoforms, p110 and p150, with different cellular functions [3, 38]. The constitutively expressed p110 isoform is localized to the nucleus and is mostly involved in nuclear RNA editing [3, 39]. The IFN-inducible p150 isoform is shuttled between cytoplasm and nuclear and is responsible in damping cytosolic dsRNA sensing by MDA5 and PKR [3, 38, 40]. Thus, we were curious as to which isoform is responsible for the increased host A-to-I RNA editing during SARS-CoV-2 infection. The p150 isoform of ADAR1 was significantly increased with levels of COVID-19 severity, while the p110 isoform showed no significant changes in low-, mid-, or high-viral load samples (Fig 2B). Moreover, the ADAR1 p150 isoform was expressed at a higher level than the p110 isoform in all viral load categories, suggesting that indeed the p150 isoform is responsible for the enhanced RNA editing in IFN-stimulated genes (Fig 2C).

### Enrichment of T cells and upregulation of ADAR1 in IFN responsive cytotoxic CD8 T cells

**Fig 3:**
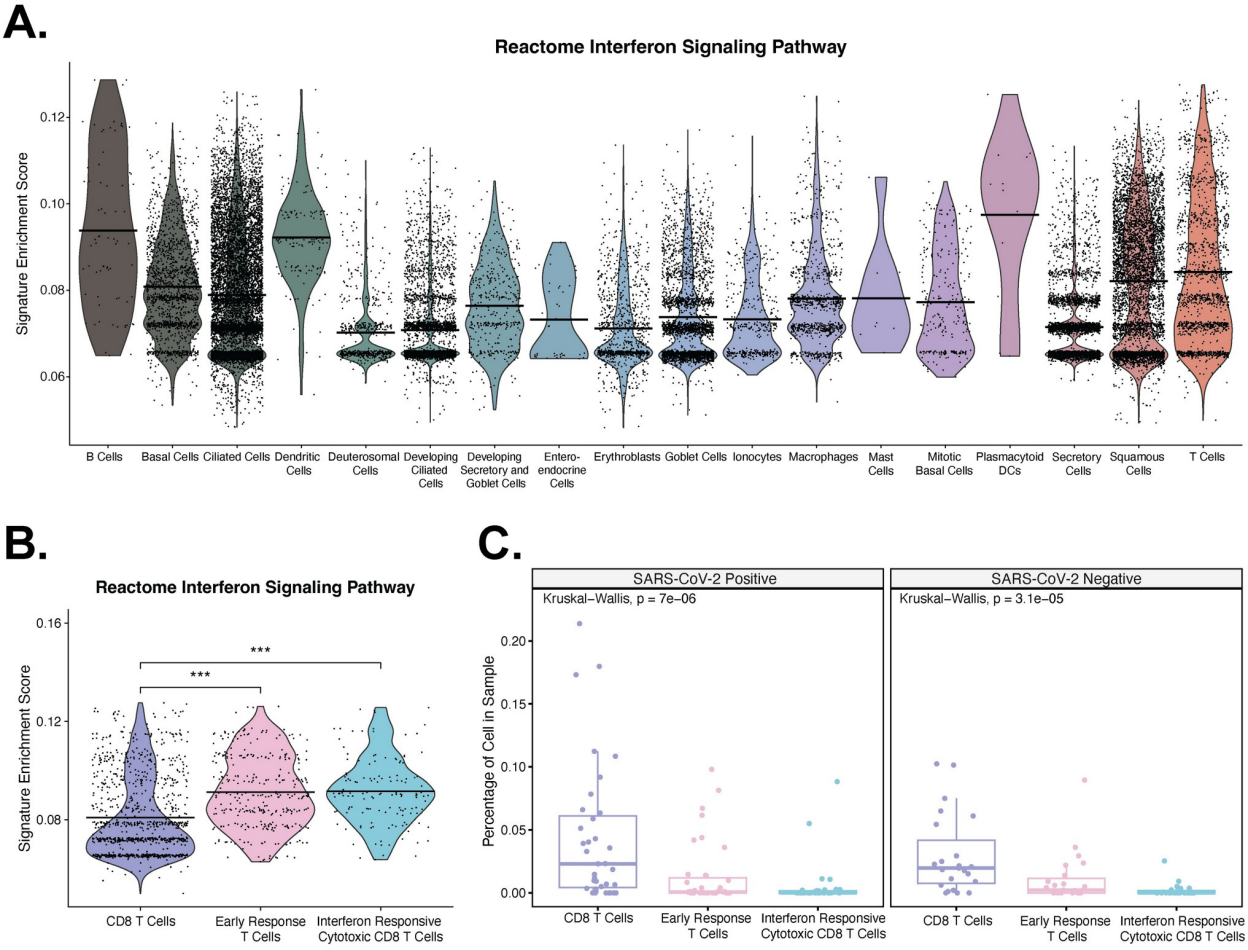
Single cell RNA-seq of SARS-CoV-2 nasopharyngeal swab samples demonstrates differences in expression of interferon signaling pathway genes and proportions. a. Expression of the Reactome interferon signaling pathway [42] among coarse cell with a Wilcox Rank Sum Test comparison. All comparisons with T cells were significant except Mast cells. b. Expression of the Reactome interferon signaling pathway [42] among T cell subtypes with a Wilcox Rank Sum Test comparison. c. Percentage of CD8, early response, and interferon responsive cytotoxic CD8 T cells in a sample, stratified by COVID-19 positive and negative samples with a Kruskal-Wallis comparison.

Next, we wanted to understand the relationship between IFNs and ADAR1 expression within SARS-CoV-2 infected immune cells. Using scRNA-seq data performed on nasopharyngeal swabs from 58 healthy and COVID-19 participants from Ziegler et al. 2021 [34], we performed UCell [41] enrichment across all cell types for Reactome IFN signaling pathway [42] to determine immune cell subtypes enriched in IFN signaling and activation in SARS-CoV-2 infection. We found enrichment in plasmacytoid dendritic cells, B cells, and T cell subtypes (Fig 3A and 3B). Out of the 1,475 cells that were previously identified as T cells [34], we found a significant increase in the enrichment of IFN signaling pathway between CD8 T cells and early response T cells and between CD8 T cells and IFN responsive cytotoxic T cells (Fig 3B). Additionally, we found that the percentage of cells in a sample is higher in CD8 T cells for both SARS-CoV-2 positive and negative samples (Fig 3C), though no difference in the percentage of cells in a sample is observed within T cell subtypes between SARS-CoV-2 positive and negative samples (S6 Fig in S1 File).

**Fig 4:**
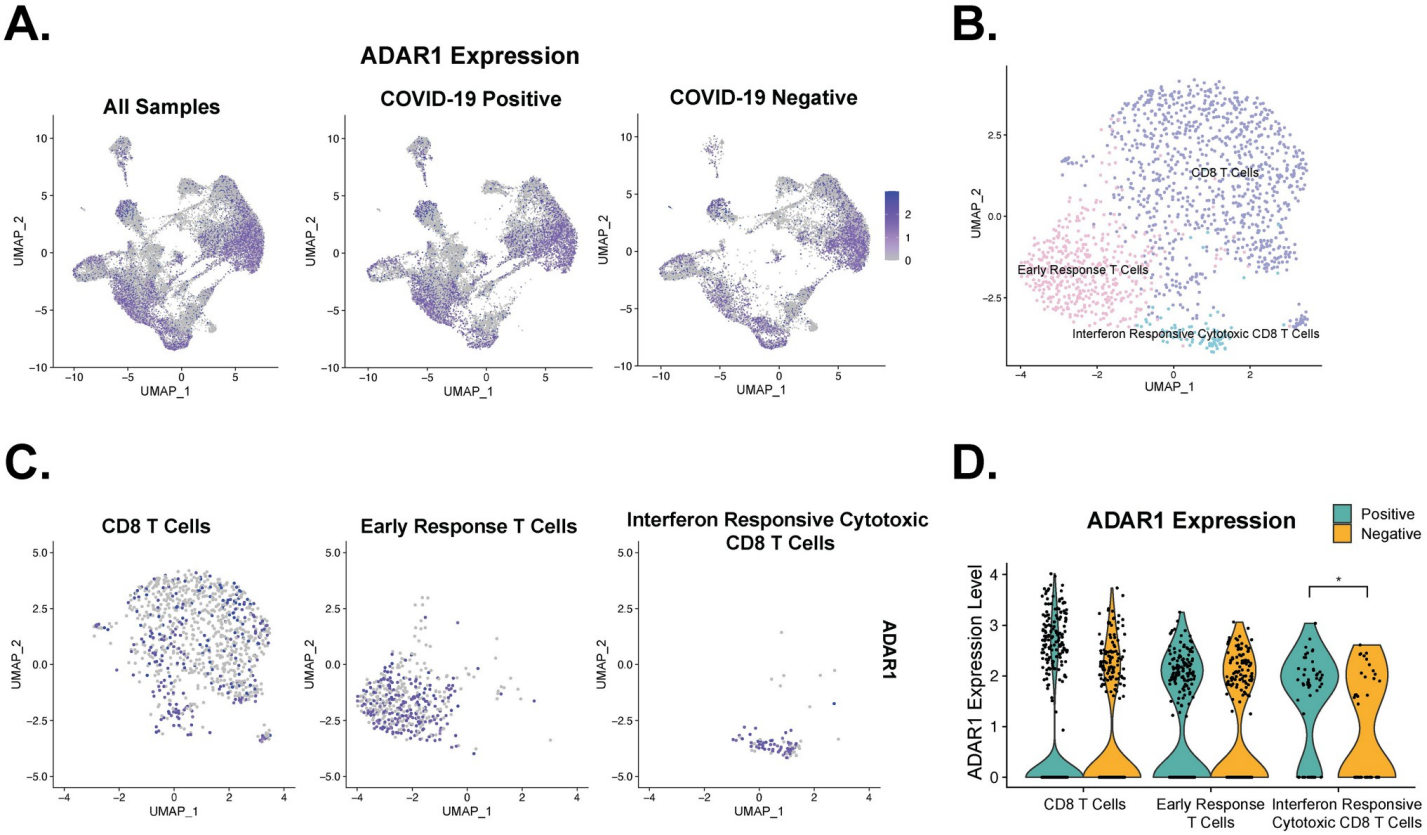
Single cell RNA-seq of SARS-CoV-2 nasopharyngeal swab samples reveals increased ADAR1 expression in interferon responsive cytotoxic CD8 T cells. a. UMAP ADAR1 expression divided by COVID-19 positivity and UMAP of ADAR1 expression across all samples. b. UMAP of T cells colored by subtypes. c. UMAP of ADAR1 expression in T cells separated by subtypes. d. Interferon responsive cytotoxic CD8 T cells differentially expressed ADAR1. * p < 0.05.

We then interrogated ADAR1 expression across all cell types using the clusters determined by Ziegler et al. 2021 and confirmed the presence of ADAR1 in all clusters (Fig 4A). Using the previously reported cell type markers [34] and Seurat integration [43], we confirmed the three distinct subtypes of T cells: Early Response T cells, IFN responsive cytotoxic CD8 T cells, and CD8 T cells (Fig 4B). We examined the ADAR1 expression within each T cell subtype (Fig 4C). Interestingly, ADAR1 was differentially expressed only in the IFN responsive cytotoxic CD8 T cells subset, with 74.5% of COVID-19 positive cells and 48.7% of COVID-19 negative cells expressing ADAR1, which likely represents an overexpression of IFN responsive ADAR1 p150 isoform (Fig 4D, p-val = 0.0269). Of note, the overall ADAR1 expression was higher in CD8 T cells than in the other two subtypes of T cells. Due to the limitation of read coverage in scRNA-seq, we are not able to decisively conclude isoform expressions in these three CD8 T cell subtypes. However, given the important role of p150 isoform in controlling dsRNA sensing and IFN response and the elevated expression in COVID positive cohort (Fig 2B and 2C), it is reasonable to assume that ADAR1 activation prevents overstimulation of IFN response in CD8 cytotoxic T cells during SARS-CoV-2 infection.

### ADAR1 p150 is overexpressed during T cell activation

**Fig 5:**
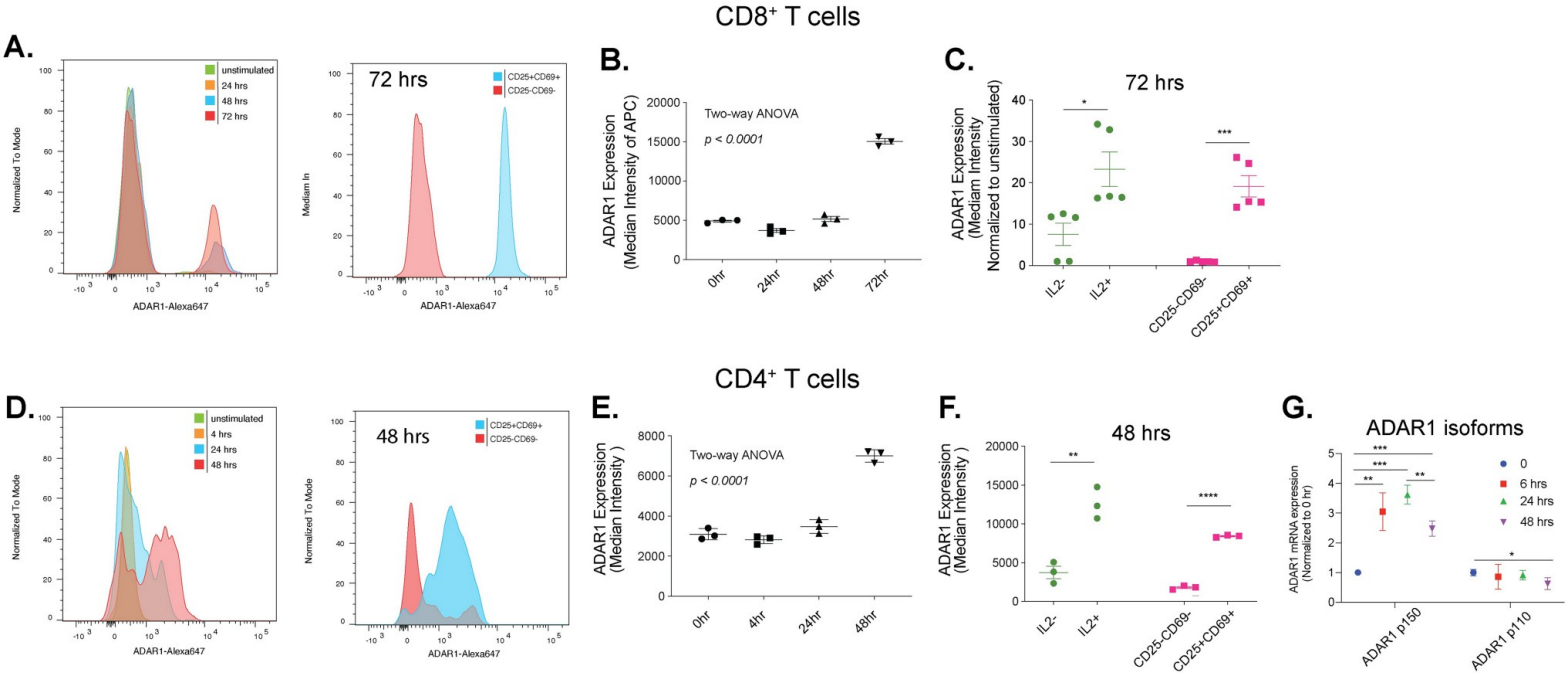
ADAR1 protein expression in CD4 and CD8 T cells attributed to ADAR1 p150 isoform. a. Representative FACS plots showing ADAR1 activation in CD3/CD28 stimulated CD8 T cells. b. ADAR1 protein expression was quantified in CD8 T cells using intracellular staining after 0, 24, 48, or 72 hours of stimulation with CD3/CD28 Dynabeads (n = 3 biological samples). Two-way ANOVA analysis. c. ADAR1 protein expression was assessed in IL2-, IL2+, CD25-CD69- and CD25+CD69+ naïve or activated CD8 T cells after 72 hours of stimulation (n = 5 samples). Unpaired student t-test. * p < 0.05; *** p < 0.001. d. Representative FACS plots showing ADAR1 activation in CD3/CD28 stimulated CD4 T cells. e. ADAR1 protein expression in CD4 T cells using intracellular staining after 0, 4, 24 or 48 hours of stimulation with CD3/CD28 Dynabeads (n = 3 biological samples). Two-way ANOVA analysis. f. ADAR1 protein expression was assessed in IL2-, IL2+, CD25-CD69- and CD25+CD69+ naïve or activated CD4 T cells after 48 hours of stimulation (n = 3 samples). Unpaired student t-test, ** p < 0.01; **** p < 0.0001. g. The gene expression of ADAR1 p150 and p110 isoforms were detected in activated CD4 T cells (n = 3 samples). Unpaired student t-test, * p < 0.05; ** p < 0.01; *** p < 0.001.

Here, we investigated whether ADAR1 p150-mediated RNA editing has any functional roles during T cell activation. We activated naïve CD4 and CD8 T cells with CD3/CD28 beads and quantified the ADAR1 protein and mRNA levels after various time points of activation (Fig 5A–5G). We observed a gradual increase in ADAR1 protein level that peaked at 72 hours (∼2.5 fold comparing to naïve cells) in CD8 T cells (Fig 5B and 5C). In CD4 T cells, a less intense (∼2-folds comparing to naïve cells) but fast activation of ADAR1 was detected at 48 hours of activation (Fig 5D and 5E). The interleukin-2 (IL2) signal and the percentage of CD25+CD69+ cells were both elevated, confirming CD8 and CD4 T cell activation (Fig 5C and 5F). Consistent with dominant activation of ADAR1 p150 isoform in SARS-CoV-2 patients (Fig 2B and 2C), increase in ADAR1 protein is mostly attributed to the ADAR1 p150 isoform rather than the p110 isoform (Fig 5G).

### ADAR1 is required for homeostasis in T cell proliferation

**Fig 6:**
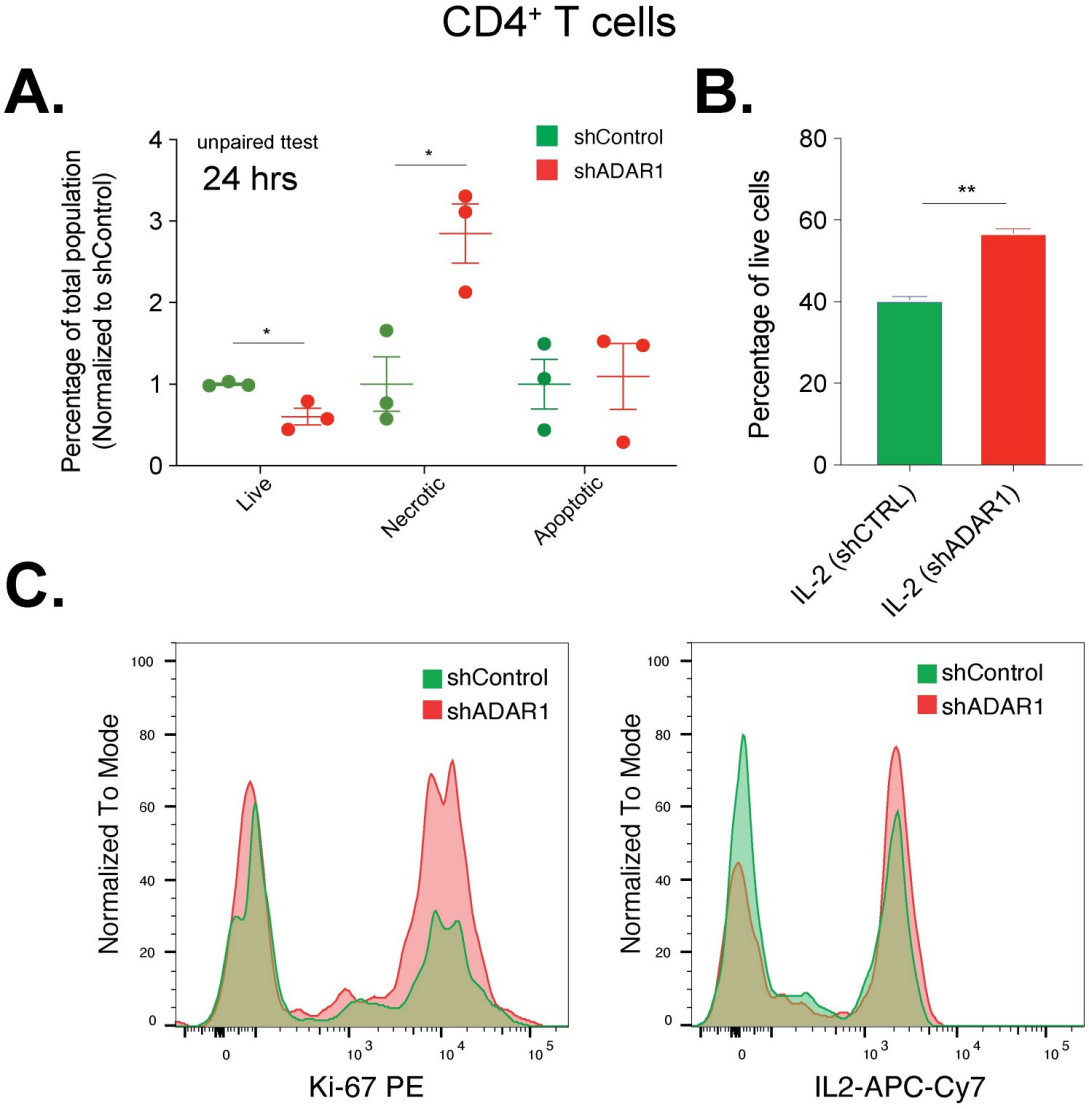
ADAR1 knockdown in CD4 T cells induces apoptosis. a. The percentages of live, necrotic, and apoptotic CD4 T cells were measured after 24 hours of CD3/CD28 stimulation. Unpaired student t-test, * p < 0.05. b. Level of IL2 was indicated in shControl or shADAR1 CD4 T cells (n = 2). Unpaired student t-test, ** p < 0.01. c. Flow plots showing increase levels of Ki-67 and IL2 in CD4 cells with ADAR1 knockdown.

ADAR1 p150 is uniquely responsible for damping IFN-stimulated apoptosis by introducing hyper-editing in ISGs [3, 44–47]. Since we observed hyper-editing in several well documented ADAR1 target ISGs (ISG15, EIF2AK2, and OASL) and IFN pathways, as well as MDA5 dsRNA sensor signaling pathway (Fig 2A and S4 Fig in S1 File) [20, 48–50], we hypothesized that ADAR1 is required to prevent apoptosis during T cell activation and positive IFN signaling. We performed lentiviral directed ADAR1 knockdown in naïve CD4 cells followed by CD3/28 activation to assess changes in cellular apoptosis (Fig 6A and S7 Fig in S1 File). ADAR1 inhibition increases necrosis and reduces the percentage of healthy T cells (Fig 6A). Interestingly, ADAR1 knockdown leads to accelerated cell proliferation, as measured by Ki-67 marker and overproduction of IL2 (Fig 6B and 6C). Therefore, we conclude that activation of ADAR1 is required to maintain a healthy homeostasis in T cell proliferation and prevent cell death by damping IFN-induced apoptosis stress.

## Discussion

Several studies have shown a link between A-to-I RNA editing and SARS-CoV-2 infection [13–16, 22, 23, 51]. Widespread A-to-I RNA editing has been detected in the SARS-CoV-2 viral genome from various types of patient biospecimens and is thought to limit infectibility by the host [14, 22, 23]. In addition, these ADAR1-induced changes also present a unique force for driving viral evolution and fitness [13–16, 22, 23, 52]. Our work confirms that ADAR1-directed RNA editing is widely detected in host immune cells during SARS-CoV-2 infection [24, 53, 54]. Differentially edited transcripts suggest a diverse T cell response between SARS-CoV-2 and OVI, due to enrichment in pathways such as T cell activation, T cell activation involved in immune response and T cell differentiation involved in immune response. Differentially expressed genes were also enriched in T cell pathways. Furthermore, differentially edited IFN signaling pathway genes were upregulated in COVID-19 positive samples and downregulated in COVID-19 negative samples.

Of note, we observed that ADAR1 p150 expression and RNA editing in ISGs increase with increased severity in viral load, while overall RNA editing reduces gradually, suggesting that efficient RNA editing in IFN genes by p150 is critical to suppress IFN signals and immune system overdrive. Merdler-Rabinowicz et al. 2023 recently examined the same dataset, and the authors showed that total ADAR1 expression is indeed increased with higher viral load [23]. In contrast, the 3’UTR-Alu editing, which is thought to represent cytosolic p150 activity, is not changed within low-, mid-, and high- viral loads. Together, these observations raise the questions of whether the reduced overall RNA editing activity represents differential targeting of the p110 isoform or differential activity of trans- and cis-regulators that controls ADAR1 functions [55]. Future studies are necessary to reveal regulatory mechanisms that control ADAR1 p150 and p110 activity in SARS-CoV-2 infection.

After finding this connection between differential editing and T cell pathways, we wanted to better characterize the relationship between IFNs, ADAR1, and T cells. We determined that the Reactome IFN signaling pathway [42] was significantly enriched in T cells compared to other cell types, which is notable as IFN signaling leads activates ADAR1 p150 isoform, which is responsible for damping IFN-induced apoptosis thereby protecting cells during IFN stress. Lastly, we find that ADAR1 expression is upregulated in IFN responsive cytotoxic CD8 T cells.

As we observed disruption of T cell activation pathways during SARS-CoV-2 infection, we wanted to determine whether ADAR1 RNA-editing is implicated in T cell activation. CD4+ and CD8+ T cells both demonstrated increases in ADAR1 p150 expression. Mechanistically, ADAR1 knockdown leads to accelerated cell proliferation coupled with necroptosis and overproduction of IL2. As such, we find that ADAR1 activation is necessary for homeostasis in T cell proliferation. Future investigation will be necessary to accurately assess the thresholds of RNA editing and ADAR1 p150 expression required for homeostasis of SARS-CoV-2 infected T cells and to determine whether the various ability of activating ADAR1 contributes, at least in part, to the diverse immune response in COVID-19 patients. Further, as elevation of ADAR1 RNA-editing has been previously observed to be transient in COVID-19 patients [23], the connection between ADAR1 RNA-editing and T cells will be relevant towards investigating whether ADAR1 RNA-editing plays a role in long COVID-19, debilitating illness as a result of COVID-19 infection [56]. Patients with long COVID-19, in particular, demonstrate increased exhausted CD4+ T cells [57], persistent cytotoxicity in CD8+ T cells [58], and persistent T cell activation [59], though long COVID-19 correlation with T cells is conflicting [60]. Characterizing the relationship between T cells and ADAR1 in SARS-CoV-2 infected samples may also lead to better prediction of patient response and disease severity.

## Materials and methods

### Resource availability

Raw sequencing files for the bulk RNA sequencing analysis were downloaded from dbGAP (accession # 38851, ID phs002258.v1.p123) [33]. Normalized counts and metadata for the single cell RNA sequencing analysis were downloaded from Single Cell Broad Institute Portal (SCP1289) [34].

All source code has been deposited at https://github.com/UCSD-Fisch-Lab/ADAR1_COVID-19 and archived source code at the time of publication is available from DOI: 10.5281/zenodo.11289138. All data required for reproducing the results of this manuscript are available as part of the article and no additional source data are required.

### Experimental model and subject details

#### Primary cell cultures

Cryopreserved CD4 and CD8 T cells were purchased from AllCells (Catalog #70015 and 70016).

### Method details

#### Bulk RNA sequencing: RNA editing analysis

Human whole transcriptome data from nasopharyngeal swabs was collected as described previously [33]. We eliminated the follow samples for conflicting designations between type of viral infection and RT PCR result: COVSUBJ_0137_1_N_HA, COVSUBJ_0146_1_N_HA, COVSUBJ_0376_1_N_HA, COVSUBJ_0461_1_N_HA, COVSUBJ_0558_1_N_HA, and COVSUBJ_0619_1_N_HA. We performed RNA editing analysis to obtain A-to-I editing events present in at least 5% of samples according to previously published methods [61]. Variants were called according to GATK4 Best Practices [62] using hg38 and filtered [61] to identify A-to-I sites in REDIportal [63]. We performed differential editing at each site using a log likelihood ratio test [64]. Enrichment analysis of differential editing genes (adj.p < 0.05) was performed using gProfiler [65]. Oncoplot was plotted using maftools [66]. Differentially expressed genes and isoforms for SARS-CoV-2 positive versus negative samples, other viral illness and viral load were obtained using the limma-voom method after TMM-normalization [67]. Violin plots, heatmaps, and barplots were plotted using the ggplot2 package (v 3.3.6).

#### Single cell RNA sequencing

Single cell RNA-sequencing data of SARS-CoV-2 infected nasopharyngeal swabs was downloaded from Single Cell Broad Institute Portal (SCP1289) [34]. The Seurat (v 4.1.0) package [43] was used to perform dimensionality reduction with the RunPCA() function with 36 principle components. Louvain clustering was utilized to cluster the cells. The same 36 principal components were used to define Uniform Manifold Approximation and Project (UMAP) and nearest neighbors. Enrichment of Reactome IFN signaling pathway was found using UCell (v 3.15) [41]. To determine the cell type proportions by sample, we used dittoBarPlot() from DittoSeq [68]. The dataset was then subset to only include samples previously identified as T cells [34]. Integration was performed using Seurat and expression of ADAR1 was found using FeaturePlot(). Conserved cell type markers as found in Ziegler et. al 2021 were used to identify the clusters [34]. Differentially expressed genes between COVID-19 positive and negative samples in cell types were compared using a Wilcox ranked sum test and VlnPlot() was used to visualize the data.

#### Human T cell activation

To activate T cells, cells were plated in 24-well plate at a density of 5x105 per mL of X-VIVO 15 media (LONZA). CD3/CD28 Dynabeads Human T cell beads (ThermoFisher) were added at 1:1 ratio as suggested by manufactory’s protocol. Cells were maintained at 37C and 5% CO2.

#### Intracellular flow cytometry

Cells (2–5x104) were aliquoted into 96-well plate and blocked with anti-human FcR blocking reagent (BD Biosciences) for 30 minutes on ice. Ethidium monoazide (EMA) was added to cells and incubated for 15 minutes in dark and then 15 minutes in bright light. After washing, cell surface antibodies were added to a final dilution of 1:25 and incubated for 45 minutes on ice in the dark. Cells were then fixed and permeabilizated with an intracellular buffer set (eBioscience) according to the manufacture’s protocol followed by staining with an antibody against ADAR1 (Abcam, ab126745) at 1:100 dilution for 30 minutes. Secondary antibody of anti-rabbit Alexa647 (Invitrogen) were added at 1:500 dilution for 30 minutes. Flow cytometry was performed on MACSQuant 10 analyzer (Miltenyi Biotec).

#### Lentiviral ADAR1 knockdown

All lentivirus were produced in HEK293T cells and the titer was determined by RT-qPCR of p24 ELISA or RT-qPCR of the 5/ LTR region. CD8 and CD4 naïve T cells were plated in 24-well plate (1x106) in X-VIVO15 media and transduced with lentivirus expression shRNA targeting ADAR1 at a MOI of 50–100 [69, 70]. Cells were collected after 2–3 days for downstream analysis.

#### Apoptosis assay

Cells were collected after 24 hours of CD3/CD38 stimulation. The magnetic beads were removed by separation on a magnetic column for 1–2 minutes. Supernatant contains activated cells for flow cytometry. Apoptosis assays were performed using PacBlue-Annxin V AADvanced Apoptosis kit (ThermoFischer, Catalog #A35136).

#### RNA extraction and RT-qPCR

Total RNA extraction was performed using RNeasy Mini Kit (Qiagen) and the RNA quantify was determined by NanoDrop. Complementary DNA was made by SuperScript III First strand synthesis system (Invitrogen) followed by RT-qPCR using SYBR GreenER qPCR supermix (Invitrogen) on CFX384 system (BioRad). 5 ng of template cDNA and 0.2 uM of each forward and reverse primer were applied. Human HPRT expression was used as housekeeping control. ADAR1 p150 primers: (fw 5’ to 3’: AAC GAA AGC GAA ATT GAA CC; rev 5’ to 3’: GGG TGT AGT ATC CGC TGA GG). ADAR1 p110 primers: (fw 5’ to 3’: GAC TGA AGG TAG AGA AGG CTA CG; rev 5’ to 3’: TGC ACT TCC TCG GGA CAC). HPRT primers: (fw 5’ to 3’: CGTCTTGCTCGAGATGTGATG; rev 5’ to 3’: TTTATAGCCCCCCTTGAGCAC).

## Supporting information

S1 File (DOCX)

S1 Table Percentage of differentially edited sites and gene name of the differentially edited sites. (XLSX)

S2 Table gProfiler pathways of differentially edited genes. (XLSX)

S3 Table Differentially expressed genes. (XLSX)
