# Supplementary material for: RNA editing regulates host immune response and T cell homeostasis in SARS-CoV-2 infection
Source: PLoS One. 2024 Aug 23;19(8):e0307450. doi: 10.1371/journal.pone.0307450 (PMC11343423; doi:10.1371/journal.pone.0307450)
Supplement: S1 File — (DOCX) [file pone.0307450.s001.docx]

**S1 Fig. Top differentially edited genes.** Top differentially edited genes based on adjusted p-value across all samples and comparisons, related to Figure 1.

A.) 11 unique genes differentially edited across all samples. B.) Top 10 most significantly differentially edited genes in the SARS-CoV-2 positive versus negative comparison. C.) Top 10 most significantly differentially edited genes in the SARS-CoV-2 positive versus other viral illness (OVI) comparison. D.) Top 10 most significantly differentially edited genes in the OVI versus negative comparison. E.) Top 10 most significantly differentially edited genes in the SARS-CoV-2 high versus SARS-CoV-2 low comparison.

**A.**

| **VarID** | **Hugo Symbol** | **Variant Classification** |
| --- | --- | --- |
| 12:122711830 | HCAR3 | 3'Flank |
| 17:1636530 | SCARF1 | Intron |
| 17:39763916 | IKZF3 | 3'UTR |
| 17:80320453 | RNF213 | Intron |
| 20:32080568 | HCK | Intron |
| 20:32080574 | HCK | Intron |
| 2:144410050 | ZEB2 | Intron |
| 4:17602764 | LAP3 | Intron |
| 6:109391124 | PPIL6 | 3'Flank |
| 8:102992837 | AZIN1-AS1 | Intron |
| 9:32497558 | DDX58 | Intron |

**B.**

| **VarID** | **COVID-19 Infection** | **No Infection** | **p.adj** | **Hugo Symbol** | **Variant Classification** |
| --- | --- | --- | --- | --- | --- |
| 4:88400238 | 0.26046512 | 0.03263403 | 1.03E-73 | HERC6 | Intron |
| 12:50930762 | 0.54418605 | 0.20979021 | 6.96E-63 | METTL7A | 3'UTR |
| 4:88399696 | 0.2372093 | 0.02797203 | 1.04E-61 | HERC6 | Intron |
| 12:112976114 | 0.18139535 | 0.004662 | 1.58E-61 | OAS3 | 3'Flank |
| 19:17401923 | 0.1627907 | 0.01165501 | 3.67E-57 | BST2 | 3'Flank |
| 9:32482751 | 0.14418605 | 0.01631702 | 2.12E-54 | DDX58 | Intron |
| 4:88404441 | 0.17209302 | 0.01631702 | 3.52E-53 | HERC6 | Intron |
| 2:6838123 | 0.14883721 | 0.01398601 | 3.43E-51 | CMPK2 | 3'Flank |
| 19:17401393 | 0.13488372 | 0.01165501 | 1.36E-50 | BST2 | 3'Flank |
| 10:89331677 | 0.2 | 0.03030303 | 3.31E-49 | IFIT3 | Intron |

**C.**

| **VarID** | **Other Viral Infection** | **COVID** | **p.adj** | **Hugo Symbol** | **Variant Classification** |
| --- | --- | --- | --- | --- | --- |
| 10:113756402 | 0.1097561 | 0.0372093 | 0.00083132 | PLEKHS1 | Intron |
| 10:12358413 | 0.15853659 | 0.03255814 | 0.00232618 | CAMK1D | Intron |
| 10:12422515 | 0.20731707 | 0.05581395 | 0.00034804 | CAMK1D | Intron |
| 10:12442370 | 0.1097561 | 0.02790698 | 0.00909029 | CAMK1D | Intron |
| 10:12578692 | 0.20731707 | 0.0744186 | 0.00659809 | CAMK1D | Intron |
| 10:125954799 | 0 | 0.06976744 | 0.00729028 | FANK1 | Intron |
| 10:128053166 | 0.25609756 | 0.08837209 | 0.00010972 | PTPRE | Intron |
| 10:22661449 | 0.14634146 | 0.04651163 | 0.00233868 | PIP4K2A | Intron |
| 10:29580765 | 0.12195122 | 0.02790698 | 0.00025574 | SVIL | Intron |
| 10:30982266 | 0.08536585 | 0.03255814 | 0.01294389 | ZNF438 | Intron |

**D.**

| **VarID** | **Other Viral Infection** | **No Infection** | **p.adj** | **Hugo Symbol** | **Variant Classification** |
| --- | --- | --- | --- | --- | --- |
| 10:89331803 | 0.30487805 | 0.02797203 | 5.27E-48 | IFIT3 | Intron |
| 4:88400238 | 0.26829268 | 0.03263403 | 9.13E-48 | HERC6 | Intron |
| 4:17602764 | 0.2195122 | 0.004662 | 1.07E-47 | LAP3 | Intron |
| 9:32482751 | 0.19512195 | 0.01631702 | 5.27E-45 | DDX58 | Intron |
| 12:112976114 | 0.2195122 | 0.004662 | 1.51E-40 | OAS3 | 3'Flank |
| 9:32497558 | 0.25609756 | 0.02564103 | 3.65E-38 | DDX58 | Intron |
| 10:89331285 | 0.19512195 | 0.02331002 | 1.60E-37 | IFIT3 | Intron |
| 13:42988984 | 0.24390244 | 0.01631702 | 1.62E-37 | EPSTI1 | Intron |
| 4:88399696 | 0.24390244 | 0.02797203 | 3.84E-35 | HERC6 | Intron |
| 21:41448003 | 0.47560976 | 0.11655012 | 4.19E-35 | MX1 | Intron |

**E.**

| **VarID** | **High** | **Low** | **p.adj** | **Hugo Symbol** | **Variant Classification** |
| --- | --- | --- | --- | --- | --- |
| 10:89331978 | 0.25806452 | 0 | 5.43E-13 | IFIT3 | Intron |
| 19:17400082 | 0.17741935 | 0 | 2.67E-11 | BST2 | 3'Flank |
| 9:32482679 | 0.24193548 | 0 | 3.46E-11 | DDX58 | Intron |
| 1:160998026 | 0.09677419 | 0 | 9.83E-11 | F11R | 3'UTR |
| 10:89331285 | 0.30645161 | 0.01923077 | 1.79E-10 | IFIT3 | Intron |
| 10:89331803 | 0.35483871 | 0.03846154 | 4.48E-09 | IFIT3 | Intron |
| 16:15411845 | 0.12903226 | 0 | 4.60E-09 | MPV17L | 3'UTR |
| 9:32456320 | 0.19354839 | 0.03846154 | 4.61E-09 | DDX58 | 3'UTR |
| 7:6751877 | 0 | 0.21153846 | 5.97E-09 | RSPH10B2 | 5'Flank |
| 3:41210821 | 0.20967742 | 0 | 2.70E-08 | CTNNB1 | Intron |

**S2 Fig. Distribution of variant classifications between SARS-CoV-2 positive and normal differential editing.** The differential editing sites between SARS-Cov-2 and normal samples are primarily intronic, related to Figure 1**.**

**S3 Fig. Differential editing and expression of genes between SARS-CoV-2 positive and normal samples.** Count of differentially edited, upregulated, and downregulated genes.


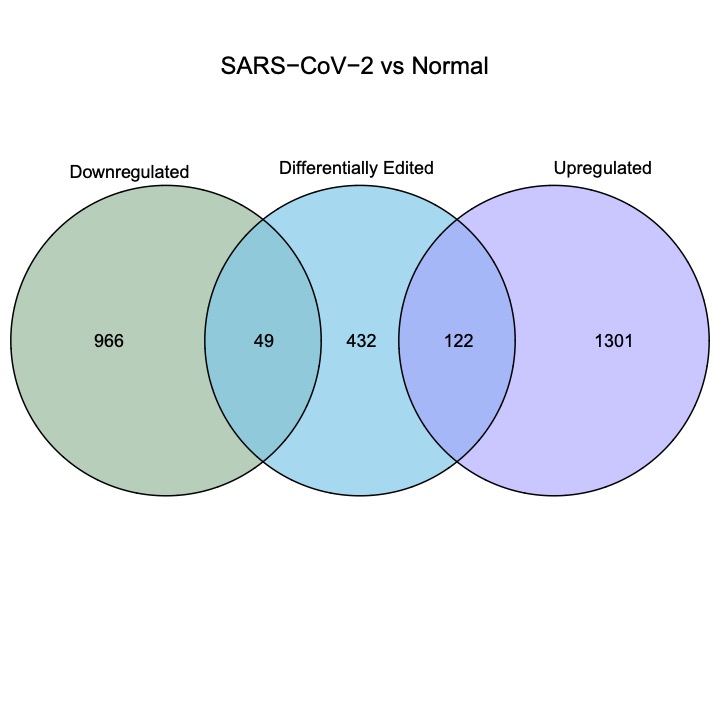


**S4 Fig. Differential expression of differentially edited genes when viral load changes in SARS-CoV-2.** Heatmap shows the upregulation (red) and downregulation (blue) of genes that were both differentially edited and involved with Reactome Interferon (IFN) signaling across all SARS-CoV-2 viral loads. Genes are ordered by hierarchical clustering while samples are ordered by none, low, medium, and high.

**S5 Fig. ADAR family gene expression change between SARS-CoV-2 positive and normal samples.** Log fold change (logFC) of the expression of ADAR family genes between SARS-CoV-2 and normal samples, related to Figure 2.

**S6 Fig. Cell type percentages in a sample divided by SARS-CoV-2 positivity.** Center log-ratio transformed percentage of CD8, early response, and IFN Responsive Cytotoxic CD8 T cells in a sample divided by COVID-19 positive and negative samples. The statistical significance of the differences between SARS-CoV-2 positive and negative samples was determined by a Mann-Whitney test, with ties. No difference is observed between CD8, early response, and IFN Responsive Cytotoxic CD8 T cells in a sample when divided by COVID-19 positivity, related to Figure 4.

**S7 Fig. Validation of flow cytometry results.** a. Pictures showing fluorescent signals from CD4 T cells after transduction with a scramble control shRNA (shControl) or shRNA targeting ADAR1 (shADAR1). Lentiviral vector was marked by EGFP marker. b. Representative flow cytometry plot showing the percentage of apoptosis, nerosis, and live cell populations of T-cells after ADAR1 knockdown, related to Figure 6.**
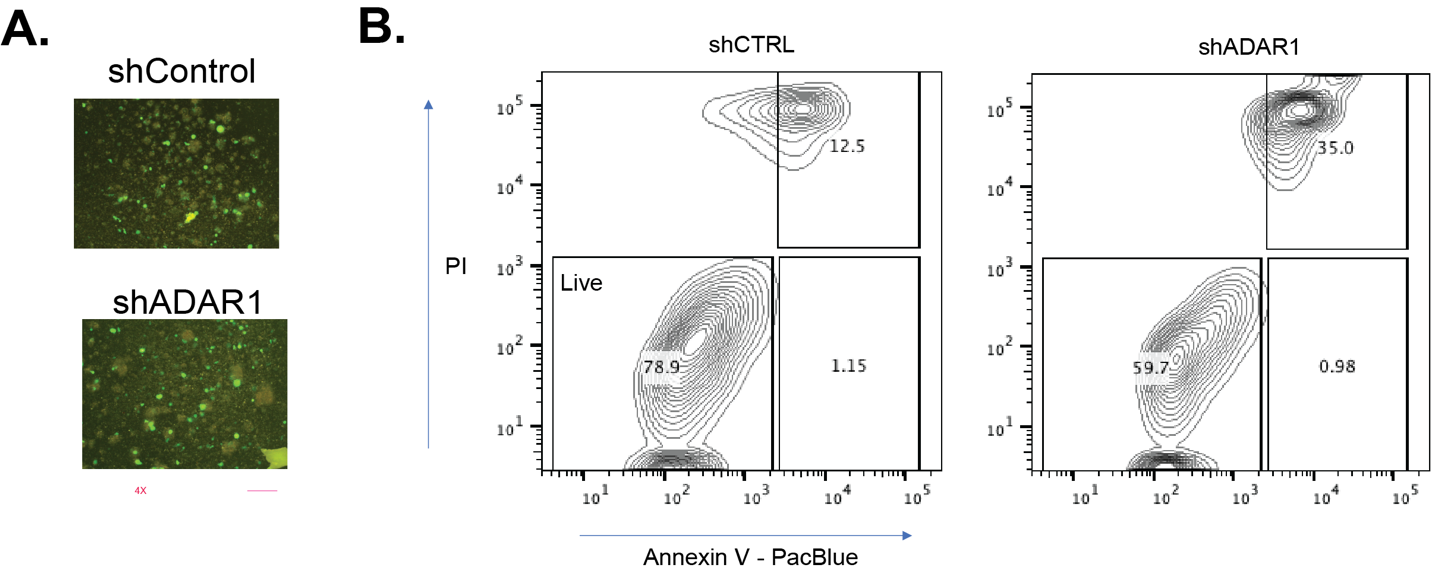
**
